# 

**Published:** 1891-03

**Authors:** 


### CONTENTS.

ORIGINAL ARTICLES—Five Cases of Bone Packing-Senn's Method—By P. O'Keef, M. D., Ononto, Wis .......... 109
Consumption—By C. A. Gill, M. D., Madison, Wis .......... 112
Under what Conditions can Electricity be of Positive Service to the Gynecologist?—By A. H. Currier, M. D., New York .......... 118
Treatment of Pneumonia in Childhood—By L. P. Barber, M. D., Tracy City, Tenn., .......... 122
Treatment of Pneumonia—By T. L. Lallerstedt, M. D., Panola, Ga .......... 125
Lindenshmidt's Urethral Irrigators and Irrigating Dilators .......... 126
A Much Needed Work—By N. B. Warren, Hazel Dell, Miss .......... 127
SOCIETY NOTES—Proceedings of the Academy of Medicine and Surgery Richmond, Va .......... 128
Gynecological and Obsterical Society of Baltimore .......... 133
Chicago Pathological Society .......... 140
BOOK REVIEWS—Twelve Lectures on the Structure of the Central Nervous System—By Dr. Ludwig Edinger .......... 146
The Second Edition of Text-Book Hygiene—By G. H. Rohe, M. D .......... 146
Encyclopedia of the Diseases of Children—By J. M. Keating .......... 147
EDITORIAL—The Medical Student and His Preceptor .......... 148
Thirty-Third Annual Commencement of the Atlanta Medical College 150
Twelfth Annual Commencement of the Southern Medical College .......... 151
Dr. Benjamin Lee—Dr. James A. Lydston .......... 154
SELECTIONS AND ABSTRACTS—The Germicidal Action of the Blood .......... 155
Salol in Typhoid Fever .......... 156
Asthma .......... 156
For Pediculi Pubis—Burns of the First and Second Degrees .......... 154
Infantile Convulsions .......... 147

